# Study on Willingness to Pay and Impact Mechanism of Gutter Oil Treatment: Taking Urban Residents in Sichuan Province as an Example

**DOI:** 10.3389/fpsyg.2021.711218

**Published:** 2021-11-11

**Authors:** Peng Cheng, Liang Xinyu, Guo Sidai, Qian Yubing

**Affiliations:** School of Economics and Management, Southwest University of Science and Technology, Mianyang, China

**Keywords:** gutter oil, willingness to pay, impact mechanism, past experience, risk perception

## Abstract

“Gutter oil” is a term for the practice of recycling used waste oil from restaurant fryers, sinks, and even slaughterhouses and sewers, and has been a major food safety and sanitation issue in China for many years. However, with proper treatment, these issues can be mitigated, turning large amounts of waste product into valuable resources and conserving energy resources. Based on this questionnaire survey conducted in the cities of Chengdu and Mianyang in Sichuan, China, this paper uses the value evaluation method to measure urban residents’ willingness to pay for the treatment of gutter oil, and explores the factors and path influencing residents’ willingness to pay based on the extended theory of planned behavior. The results of this study affirms the validity and universality of the Theory of Planned Behavior. Behavioral attitude, perceived behavioral control, and subjective norms have a direct positive impact on their willingness to pay. Risk perception and past experience indirectly affect residents’ willingness to pay for gutter oil through the intermediary variable of behavioral attitude, which means that the public’s risk awareness can be improved by vigorously publicizing the harmful effects of gutter oil, thereby also increasing acceptance toward gutter oil treatment. As an intermediary variable, subjective norms have a significant indirect effect on the impact path of past experience on willingness to pay, which reflects the significant influence of subjective norms such as reference group and environment. The results show that urban residents have a higher willingness to pay for the treatment of gutter oil. The mean willingness to pay is 7.75 RMB per month per capita.

## Introduction

With the significant improvement of living standards throughout China, the thriving of China’s restaurant industry, and expansive growth of dining establishments throughout urban areas, Chinese families are paying more attention to their quality of life, and consuming more as well. As a result of all these factors, kitchen waste oil is increasing, as is concern for its treatment and quality. As a major consumer of edible oil, China produces and consumes more than 20 million tons of gutter oil every year, and the impact of this has created major food safety problems in China. These waste edible oils contain high volumes of toxins and carcinogens, and this can cause great harm to the human body (Bai, 2010; Wang and Lu, 2012). Although returning gutter oil to the dinner table presents a number of ethical and sanitation issues, its utilization value is very high. Given these factors, the most optimal possible solution may be to properly recycle gutter oil and extract the greatest possible value therefrom (Zhang et al., 2012). This not only helps prevent the sanitation issues associated with waste cooking oil, it also helps alleviate the shortage of energy resources.

Managing gutter oil incorporates issues of both waste management and food safety (Karmee, 2017). Its sustainable utilization provides a significant step toward the formation of a healthy society. About 1.8 billion liters (476 million gallons) of biofuels can be produced from gutter oil produced in China every year. Learning to sustainably utilize gutter oil is therefore very important. The production and sale of illegal cooking oil is the main obstacle to utilizing this gutter oil to produce sustainable and clean biodiesel. The government should adopt policies to encourage the legitimate and effective recycling of gutter oil, establish standards for edible oil, and implement strict inspection measures (Cho et al., 2015). It is also necessary to strengthen the government’s regulatory responsibility, improve the management and supervision system for gutter oil, and to improve the technical inspection standards of edible oil (Ramos et al., 2013; Yano et al., 2015; Wallace et al., 2017). Governments at all levels across China have expressed great concern for the recycling and treatment of illegal cooking oil. They have therefore formulated a number of policies and measures to regulate the disposal, collection, and transportation of kitchen waste, management systems thereof, and are actively carrying out special rectification activities for illegal cooking oil to combat actions in violation of these statues. By designating pilot cities for exploratory policies, they will continue to experiment with recycling, no-harm treatment methods, process routes and management modes for kitchen waste. However, it is difficult for the government to supervise waste edible oil due to highly decentralized and dispersed nature of the sources (i.e., potentially every household and restaurant in China). For example, although the Chinese government actively promotes the conversion of gutter oil into biodiesel through government regulation, subsidies, and tax incentives, issues of sourcing and the illegal sale of gutter oil have yet to be resolved (Zhang et al., 2012), which means that China’s recycling industry chain of gutter oil has not been fully formed, resulting in a desperate lack of capacity in zero-harm treatment and recycling of gutter oil. At present, problems such as irregular discharge, recycling, production, and sale of illegal cooking oil have yet to be checked. Covert transactions of illegal cooking oil still exist, and illegal cooking oil is still discovered on Chinese dinner tables from time to time.

One key factor in the gutter oil supply chain, households’ or residents’ willingness to pay for the treatment of gutter oil, can determine the recycling rate of gutter oil in China through conduction mechanisms. Financial support for gutter oil treatment is a crucial factor (Escobar et al., 2015), and financial subsidies from the government offer is an especially important measure to solve issues related to gutter oil (Zhang et al., 2014; Eguchi et al., 2015; Maria et al., 2016). Based on game theory, this paper studies the influence of tax preferences, raw material subsidies, sales subsidies, and investment subsidies for waste cooking oil (WCO) on biodiesel supply. The results shows that the price subsidy for raw materials and sales subsidy for finished products can improve the profits of biofuel enterprises and recyclers. However, existing research often ignores the role and participation of households, which is the main source of gutter oil creation. Compared with mandatory and normalized recycling performed by restaurants and other food service enterprises, the sources of domestic waste cooking oil are even more scattered, and their means of production and collection are cruder. Therefore, the recycling and management of domestic waste cooking oil is more difficult than conventional waste oil recycling. According to the principle of “the polluter pays,” it is of great practical significance to study residents’ willingness to pay for gutter oil recycling and the influencing factors thereof, since families are the primary source of gutter oil. According to a survey from Petaling, Malaysia, families have a relatively high willingness to accept (WTA) and willingness to collect and recycle waste cooking oil (WCO). The average WTA amount of households was found to be 0.72 MYR per kilogram, and the major influencing factors on this value were income level, age, education level, bidding practices, and gender of the participant, and cash incentives were found to effectively improve the participation rate of families in the collection of gutter oil (Yacob et al., 2015). Awareness and attitude are important factors that affect families’ participation in gutter oil recovery. Although many families recognize the importance of gutter oil recovery, only a few actually participate in it (Kabir et al., 2014). A survey from South Korea showed that waste cooking oil (WCO) has become the main raw material of the biodiesel industry in South Korea. Under the precondition of providing incentives, the household recovery of WCO can be improved, but this will not significantly increase the raw material production of biodiesel (Cho et al., 2015).

Urban residents are the main providers of treated gutter oil, and the academic research on this subject focuses on means of behavior analysis. As academic disciplines broaden and integrate and society continues to complexify, the classic Theory of Planned Behavior proves unable to fully interpret or accurately judge the behavioral intention of individuals. As an example of more complex theory, Fishbein theory believes that consumers’ degree of environmental cognition affects their willingness to pay and the amount they are willing to pay to a certain extent (Rodríguez-Ortega et al., 2016). Theories of cognitive psychology and cognitive behavior systematically analyze individual cognition from different angles and agree that individual cognition of given issues plays a particularly important role on an individual’s behavioral intention and indirectly affects their behavioral decision-making. A number of researchers (Carlsson, 2000; Hossain, 2003; Vassanadumrongdee and Matsuoka, 2005; Spash, 2006), respectively, analyzed individuals’ willingness to pay for services such as increased air quality, genetically modified food, and treatment air pollution. The results of these studies show that age, gender, education, occupation, income, and residence will affect consumers’ willingness to pay. However, no scholars have yet studied the willingness of urban residents to pay for the treatment of gutter oil.

Therefore, this study uses the value evaluation method to calculate the willingness to pay for the treatment and recycling of gutter oil among residents in China’s pilot cities experimenting in resource utilization and zero-harm treatment of kitchen waste. Based on the extended theory of planned behavior, this paper studies the factors influencing residents’ willingness to pay and discusses the key influencing factors of the residents’ willingness to pay by using the structural equation model. The goal of this is to provide some decision-making references for governments to formulate targeted incentive policies.

The remainder of this paper is arranged as follows. The second section mainly introduces the theoretical basis of the model, and puts forward corresponding hypotheses on the basis of theoretical and literature analysis. The third section mainly discusses the employed methods. The fourth section verifies the relevant hypotheses, and uses the Bootstrap method to further test the mediating effect of the model before obtaining the final empirical analysis results. The fifth section discusses the research results, and the sixth section briefly discusses the limitations of the research and future research directions.

## Extended Theoretical Framework of Planned Behavior and Hypothesis Development

### Extended Theoretical Framework of Planned Behavior

The theory of planned behavior is developed on the basis of the theory of rational behavior (Ajzen, 1985). The theory of rational behavior holds that individual behavior is controlled by one’s own will and driven by one’s own subjective will (Fishbein and Ajzen, 1975). However, subsequent research found not many external factors in addition to one’s own personal will have an impact on individual behavior. Therefore, the control factors of perceptual behavior are introduced on the basis of the theory of rational behavior. Perceived behavioral control refers to how many obstacles an individual will suffer from the implementation of the behavior before implementing said behavior. Therefore, the difficulty of completing the transaction, including controllability and self-efficacy. The theory of planned behavior emphasizes that three factors, namely behavioral attitude, subjective norms and perceived behavioral control, all of which affect people’s behavioral intentions, and thus their behavioral decision-making.

The theory of planned behavior has been widely used in both the fields of consumption behavior and environment (Wang et al., 2016; Wong et al., 2020). However, some scholars still believe that individual behavior is not only determined by factors such as behavioral attitude, subjective norms and perceived behavior, but also by factors such as moral norms, personal responsibility, and convenience (Nigbur et al., 2011; Wang et al., 2011; Yin et al., 2014). Ajzen also indicates that this theory is open and extensible. Scholars can also therefore introduce other theories into the theory of planned behavior according to their research needs. Therefore, based on the theoretical framework of planned behavior, this paper introduces two important influencing factors, namely, past experience (Henk et al., 1998; Forward, 2009) and risk perception (Taylor, 1974; Covello et al., 2001) to form an extended framework of Theory of Planned Behavior.

### Hypothesis Development

#### Willingness to Pay

The concept of willingness to pay (WTP) was first put forward by Hicks’ neoclassical economic theory, and is defined as the sum of consumer surplus and actual payment. When Ciriacy-Wantrup (1947) measured the positive externalities of soil erosion control, the conditional value method was first used to calculate the price that people are willing to pay for soil pollution control. Different scholars have given different analyses of these factors and definition of key terms. Kim et al. (2009) says that the WTP refers to the price that consumers voluntarily pay out for products when they participate in market transactions. Mankiw (2011) adds the definition of WTP as being the consumers’ highest psychological price. WTP is widely used in assessing public services, mainly to measure payments that the consumer is willing to pay for public services or products. This includes willingness to pay for the protection of public land (Rosenberger and Richard, 1997; Rolf et al., 1999), willingness to pay for air quality improvement (Liu et al., 2017; Wang et al., 2018), willingness to pay for treatment of water pollution (Zhao et al., 2018), and willingness to pay for soil pollution prevention (Lu et al., 2018). However, in the field of waste management, most literature focuses on public awareness of the collection and recycling of solid waste, such as urban electronic waste (Keramitsoglou and Tsagarakis, 2013; Kumar, 2019), and has rarely been concerned about the willingness to pay for gutter oil treatment.

#### Past Experience

Some scholars have confirmed that past experience has an impact on behavioral habits. Henk et al. (1998) conducted a study on “repetitive behavior” and confirmed that existing behavior is largely influenced by past behavior. Through research, Gardner (2009) confirmed that when a decision-making environment tends to be stable, past experience has a positive impact on behavioral intention. By studying a person’s primary behaviors, it is found that past experience will affect behavioral attitude and subjective norms, thus affecting individual behavioral intention (Forward, 2009). Therefore, this paper introduces the variable of past experience to study the influence of past experience on the willingness to pay for gutter oil treatment, and puts forward the following hypotheses:

H1a: The past experience of urban residents has a direct and significant positive effect on their risk perception.
H1b: The past experience of urban residents has a direct and significant positive effect on their behavior and attitude.
H1c: The past experience of urban residents has a direct and significant positive effect on their subjective norms.
H1d: The past experience of urban residents has a direct and significant positive effect on their willingness to pay.

#### Risk Perception

In the field of enterprise decision-making, studies of consumers’ modes of response to risk perception have confirmed that risk perception can significantly affect an individual’s behavioral attitude and behavioral intention, and thus can play a decisive role on individual behavior (Taylor, 1974). In the study of the public’s risk perception and response to viral distribution, it was found that the degree of risk perception will affect the individual’s enthusiasm to make a response (Covello et al., 2001). Simsekoglu (2019) established an extended theory of planned behavior including risk perception, and verified that risk perception could affect consumer purchasing behavior regarding electric vehicles. According to the experience of previous scholars, when introducing risk perception into the theory of planned behavior, the following hypotheses can be proposed:

H2a: The risk perception of urban residents has a direct and significant positive effect on their behavior and attitude.
H2b: The risk perception of urban residents has a direct and significant positive effect on their subjective norms.
H2c: The risk perception of urban residents has a direct and significant positive effect on their perceived control behavior.
H2d: The risk perception of urban residents has a direct and significant positive effect on their willingness to pay.
H2e: Risk perception plays an intermediary role on urban residents’ past experience on their willingness to pay.

#### Behavioral Attitude

Behavioral attitude is a determinant of the person’s willingness to pay (Taufique et al., 2017). Behavioral attitude is interpreted as the degree of concern and recognition of the respondents as concerns their behavior results. Positive attitudes may increase their willingness to pay, while negative and disapproving attitudes may lead to adverse intentions and behaviors (Greaves et al., 2013). If consumers are increasingly concerned about environmental sustainability, this naturally suggests they will behave in a more pro-environmental way (Wong et al., 2020), which may result in a higher willingness to pay for environmental services. This is so say that there is a significant positive link between behavioral attitude and behavioral intention (Chan et al., 2016). The more positive of an attitude that consumers have toward organic food, the more likely they will be to buy low-carbon products with carbon label certification; that is, the stronger their willingness to buy will be (Trivedi et al., 2018). However, some scholars believe that the proportion of behavioral attitude to corresponding willingness to pay is generally low. Because willingness to pay implies that payment behavior has not yet taken place, the respondents may substitute actual feelings or willingness with the response tendency to meet social expectations, and their willingness to pay will be overestimated in surveys (Roozen and Pelsmacker, 1998). Davies et al. (2002) found that 84 percent of consumers who do not participate in recycling still claim to have recycled some of their household waste. Therefore, the following assumptions are proposed:

H3a: The behavioral attitudes of urban residents have a direct and significant positive effect on their willingness to pay.
H3b: Behavioral attitudes play an intermediary role on the impact of risk perception on residents’ willingness to pay.
H3c: Behavioral attitudes play an intermediary role on the influence of past experience on Residents’ willingness to pay.

#### Subjective Norms

According to the theory of planned behavior, subjective normative factors have a positive impact on the individual’s behavioral intentions, thus affecting the occurrence of their actual behavior (Ajzen, 1991). Based on the theory of planned behavior, Yadav and Pathak evaluated the influencing factors of Indian consumers’ purchases of green products, and found that social norms can significantly affect consumers’ intent to purchase green products (2017). However, the occurrence of individual behavior is not an independent process of individual choice, and is instead often affected by social factors such as external environment and reference groups (Blaise et al., 2018). For example, social pressure is a key factor affecting the intent to recycle (Singh et al., 2018). Therefore, risk perception, past experience, and other external environmental factors will affect the willingness to pay through subjective norms. Therefore, the following hypotheses are put forward

H4a: Subjective norms of urban residents have a direct and significant positive effect on their willingness to pay.
H4b: Subjective norms play a mediating role on the impact of risk perception on willingness to cope.
H4c: Subjective norms mediate the effect of past experience on residents’ willingness to pay.

#### Perceived Behavioral Control

The theory of planned behavior emphasizes that individual perceived behavioral control has a significant impact on individual behavior. If the individual has a higher degree of controllability and self-efficacy in a risk-prone field, they are likely to implement said behavior; if the individual has a lower sense of controllability and self-efficacy in a risk-prone field, they will avoid the related behavior (Ajzen, 1991). Individuals are constrained by internal and external conditions, and this may affect their confidence in the implementation of certain behaviors. This factor is called perceptual behavioral control. This can measure the perception of individuals to certain behaviors that may be either easy or difficult to perform, as measured through a series of controlled variables that can be traditionally perceived (Tonglet et al., 2004). Research in the field of recycling behavior shows that respondents who are confident in “how, when, and where” to recycle tend to show a stronger willingness to recycle than those who believe they have existing or imminent restrictions and limited controls on their recycling behavior (Kumar, 2019). The following hypotheses are thereby proposed:

H5a: The perceived behavioral control of urban residents’ has a direct and significant positive effect on their willingness to pay for the treatment of gutter oil.
H5b: Perceived behavioral control plays a mediating role on the impact of risk perception on residents’ willingness to pay for treatment of gutter oil.

**FIGURE 1:**
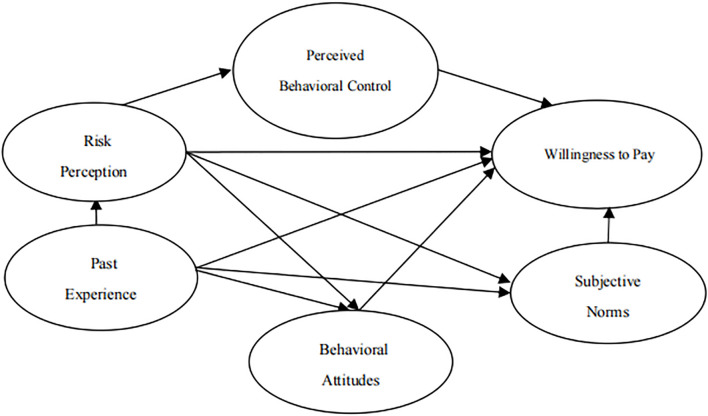
Research hypothesis model.

According to the above research hypotheses, the research hypothesis model of urban residents’ willingness to pay for treatment of gutter oil is established, as shown in Figure 1.

## Methodology

### Research Design

This paper adopts the contingent valuation method (CVM) to evaluate residents’ willingness to pay for the treatment of gutter oil. Based on the questionnaire survey, the respondents were guided to answer whether they were willing to pay a certain amount for the treatment of gutter oil. If they were willing, they were asked the specific amount they were willing to pay. Through this mode of inquiry, the average willingness to pay could be obtained from the sample subjects.

The questionnaire was designed according to the validation practices of the extended influence behavior questionnaire produced by Yukl et al. (2008). In this paper, 167 questionnaires were distributed across Chengdu and Mianyang for preliminary investigation. A total of six factors were extracted in the exploratory factor analysis, and the interpretation rate of the cumulative variance was 73.11%, indicating that the questionnaire was well-designed. In the confirmatory factor analysis, some items with high correlation values were deleted, and the ultimate model of “urban residents’ willingness to pay for gutter oil treatment” contained six latent variables (with five independent variables and one dependent variable) and 18 explicit variables, and the overall fitness of the model was found to be good. The final questionnaire included 21 research questions, three situational experimental questions and, four socio-economic background questions. The research questions were all based on a five-point Likert scale. The selection of the sample source for this study was mainly performed for three reasons. First of all, the cities of Chengdu and Mianyang in Sichuan are both pilot locations for resource utilization and zero-harm treatment of kitchen waste in China. Secondly, Sichuan cuisine is one of China’s primary culinary traditions, and many of its dishes utilize high volumes of oil. Last but not least, Sichuan is the largest center of pig breeding among any province in China, slaughtering 65.791 million pigs annually, and its waste oil from meat processing is therefore also very sizable. Therefore, Chengdu and Mianyang in Sichuan provide fertile opportunities and an ideal representative sample source.

This questionnaire survey adopts a remote structured questionnaire method. We selected four of the 28 county-level administrative regions in Chengdu and Mianyang cities as the questionnaire distribution points by simple random sampling. A total of 468 questionnaires were distributed across Chengdu and Mianyang. 431 valid questionnaires were retrieved, making for a recovery rate of 92.1%. In order to make the sample more representative, a stratified sampling method was adopted for Chengdu and Mianyang. On the basis of considering the uniform distribution of survey sites across geographical location, 10 survey sites were selected in both core urban regions and suburbs according to the principle of random sampling. The sample size of each geographical urban area is basically proportional to the local population. Before issuing the formal survey, the researchers were trained on the contents of the questionnaire, survey skills, and survey arrangements, and 60 questionnaires were pre-surveyed.

The descriptive statistical results are shown in Table 1. It can be seen that women accounted for the majority of the respondents (61.5%), basically ranging in age between 30 and 50 years old, the majority being 41–50 years old. Bachelor’s degree holders formed the largest proportion by education level, followed by those with some college, senior high school, junior high school or below, and master’s degree holders or higher respectively. The average monthly household income of the respondents was less than 20,000 yuan.

**TABLE 1:** Descriptive statistics of participants.

**Demographic characteristic**		**Sample distribution (%)**	**Frequency**
Gender	Male	38.5%	166
	Female	61.5%	265
Age	<30	24.8%	107
	31–40	25.3%	109
	41–50	39.4%	170
	51–60	6.7%	29
	>60	3.8%	16
Educational background	Junior high school or below	10.2%	44
	Senior high school	21.8%	94
	Some college	23.0%	99
	Bachelor’s degree	36.0%	155
	Master’s degree or higher	9.0%	39
Monthly household income (RMB)	<9,999	45.5%	196
	10,000–19,999	30.9%	133
	20,000–29,999	11.1%	48
	30,000–49,999	7.2%	31
	>50,000	5.3%	23

### Statistics of Willingness to Pay

This paper used a bidding card system to explore residents’ willingness to pay. Firstly, it asks whether the residents were willing to pay for the treatment of gutter oil. If not, the bidding value was given as zero. The respondents who choose “willing” were asked to choose from the given bid values, and the final statistical results of this are shown in Table 2. From the 431 questionnaires, 341 respondents were willing to pay for the treatment of gutter oil, accounting for 79.1% of the total, and 90 respondents were not willing to pay for the treatment of gutter oil, accounting for 20.9%. From the perspective of statistical proportion, most respondents stated a relatively low willingness to pay, accounting for 80.1% of the total respondents, and their willingness to pay for treatment of gutter oil was mainly between 0 and 10 yuan.

**TABLE 2:** Results of willingness to pay.

**Price range**	**Frequency**	**Percentage**
0	90	20.9%
2	69	16%
4	18	4.2%
6	27	6.3%
8	25	5.8%
10	116	26.9%
12	10	2.3%
14	2	0.5%
16	2	0.5%
18	4	1%
20	68	15.6%

Secondly, the respondents’ average willingness to pay was calculated as follows:


(1) $$\text{E}\,{\left(\mathrm{WTP}\right)}_{+}=\frac{\sum_{i}^{20}{\text{A}}_{i}\,{\text{N}}_{i}}{\text{N}}$$

Where *Ai* is the specific bid value and *Pi* is the probability of selecting the bid value. According to the statistical results, the average value of *E* (WTP) was 9.8 yuan. Note that the existence of samples willing to give zero payment increases the difficulty of this calculation, but if the zero payment samples were directly screened out, the authenticity of the results would be reduced. Therefore, on the basis of calculating the positive willingness to pay, the spike model proposed by Kristrom was used to perform adjustment (Kristrom, 1997). The core idea of this model emphasizes that if there is a zero payment sample in the tender value, the overall mean value is multiplied by the tender probability of positive payment samples on the basis of the positive payment sample’s *E* (WTP), namely,


$$\text{E}\,\left(\mathrm{WTP}\right)=\text{E}\,{\left(\mathrm{WTP}\right)}_{+}*\left(1-{\text{P}}_{0}\right)$$

According to the above formula, the final WTP is 7.75 yuan.

## Results Analysis

First of all, the possible influence of common method variance were designated to be excluded, and the scale’s reliability, aggregate validity, and discriminant validity were tested, and the degree of fit for both the data and model had to be verified. Secondly, we verified the significance of the casual relationship and the path coefficient between the variables based on the structural equation model. The indirect effect of specific variables of each mediation path was then further analyzed by the Bootstrap method.

### Measurement Validation

#### Common Method Variance

Since the collation and summary of the questionnaire data were carried out by the same person, after the implementation of the questionnaire survey, the issue of common method variance (CMV) may occur. In this paper, Harman’s single-factor method is used to test whether the common method variance is severe. Based on the software analysis of SPSS19.0, it was found that the characteristic values of the six factors were all higher than 1, thereby explaining 80.3% of the total variance. At the same time, the variance explanation rate of the first factor was 28.2%, and this is significantly lower than the critical value of 40%. This shows that common method variance is very small, and is not expected to significantly affect the research results (Harman, 1967).

#### Reliability and Validity

In this paper, we use Cronbach’s coefficient to analyze the reliability of the scale. Generally speaking, a Cronbach’s α reliability value above 0.7 is considered acceptable (Bagozzi, 1981; Nunnally and Bernstein, 1994). Table 3 shows that the coefficients of all variables were greater than 0.7, which means that the internal consistency of the questionnaire was good, and the scale possesses high reliability (Chin and Marcoulides, 1998). Validity covers both polymeric validity and discriminant validity. Polymeric validity generally requires three conditions. The first involves all factors producing a significant positive load on variables above the level of 0.001 (Lin and Niu, 2018). The second requires that the factor load should be in the range of 0.5–0.95, and the third needs the average variance extracted (AVE) to be greater than 0.5 (Fornell and Larcker, 1981).

**TABLE 3:** Reliability and convergent validity.

**Construct**	**Item**	**Item loading**	**CR**	**Cronbach’s Alpha**	**AVE**	**Skewness**	**Kurtosis**
Perceptual behavior control	PBC1	0.839				–1.597	1.775
	PBC2	0.843	0.878	0.878	0.706	–1.053	–0.032
	PBC3	0.838				–1.300	0.679
Risk perception	RP1	0.903				–1.616	2.085
	RP2	0.893	0.930	0.930	0.815	–1.986	3.648
	RP3	0.912				–2.742	7.404
Past experience	PE1	0.825				0.542	–0.380
	PE2	0.780	0.861	0.859	0.673	0.150	–0.797
	PE3	0.855				0.588	–0.441
Behavioral attitude	BA1	0.696				–1.271	1.140
	BA2	0.863	0.830	0.821	0.620	–0.631	–0.337
	BA3	0.795				–1.810	2.744
Subjective norms	SN1	0.778				–0.309	–0.985
	SN2	0.835	0.865	0.864	0.681	–0.154	–1.005
	SN3	0.860				–0.266	–0.952
Willingness to pay	WTP1	0.795				–0.434	–1.158
	WTP2	0.841	0.833	0.840	0.626	–0.215	–1.163
	WTP3	0.733				1.438	0.067

*CR, composite reliability; AVE, average variance extracted.*

Table 3 shows that all factor loads were in the interval, the AVE was significantly greater than 0.5, and the combined reliability (CR) between the factors was greater than 0.7. Therefore, the aggregate validity passes the standard.

The discriminative validity needs to compare the size relationship between the square root of the AVE and the Pearson correlation coefficient between the variables (Park et al., 2014). Table 4 shows that the square root of the AVE between each variable and its factor is greater than the Pearson correlation coefficient between variables, which means that all variables have good discriminant validity (Fornell and Larcker, 1981; Hair et al., 1998).

**TABLE 4:** Discriminant validity: AVE and Pearson correlation coefficient.

	**BA**	**PBC**	**SN**	**RP**	**PE**	**WTP**
Behavioral Attitude (BA)	**0.787**					
Perceived Behavioral Control (PBC)	0.190	**0.840**				
Subjective Norms (SN)	0.175	0.173	**0.880**			
Risk Perception (RP)	0.284	0.126	0.063	**0.903**		
Past Experience (PE)	0.188	0.090	0.141	0.308	**0.820**	
Willingness to Pay (WTP)	0.440	0.339	0.289	0.244	0.261	**0.791**

*The bold values mean that the average values are greater than relevant coefficient values, and there is sufficient discriminant validity among all dimensions.*

#### Model Fit

Amos software is used to verify the degree of fit between the questionnaire data and the hypothetical model. The results of the model fit are shown in Table 5. The results show that in the absolute fit results, the chi square value and freedom ratio is 3.142, which is less than the critical value of 5, and meets the fit standard (Carmines and McIver, 1981). The GFI is 0.907, which is greater than the critical value of 0.9, and the critical value of AGFI, close to 0.9 is acceptable. The RMSEA is 0.071, which is in the range of 0.05–0.08, and also meets the fit standard (Browne and Cudeck, 1993). In the value-added fitness results, the RFI was 0.895, which is very close to the critical value. Except for RFI, the other results all met the fit standard. This shows that there is a good fit between the model and the data.

**TABLE 5:** Results of model fit.

	**Fit indices**	**Judging criteria**	**Test result**	**Judgment**
Absolute Fit Index	CMIN/DF	<5.0	3.142	Acceptable
	GFI	>0.9	0.907	Good
	AGFI	>0.9	0.871	Acceptable
	REMSEA	<0.08	0.071	Acceptable
Value-added Fitness Index	NFI	>0.9	0.915	Good
	IFI	>0.9	0.941	Good
	RFI	>0.9	0.895	Acceptable
	TLI	>0.9	0.926	Good
	CFI	>0.9	0.940	Good

### Hypothesis Test Results

The maximum likelihood estimation method is used to test the hypotheses of the structural equation model, and the test results are shown in Table 6. It can be seen that the past experience has a significant impact on risk perception (β = 0.430, *P* < 0.001), behavioral attitude (β = 0.145, *P* < 0.01), subjective norm (β = 0.181, *P* < 0.01) and willingness to pay (β = 0.140, *P* < 0.01). Hypotheses H1a, H1b, H1c, and H1d were therefore all demonstrated to be all valid.

**TABLE 6:** Hypothesis test results.

**Hypothesis**	**+/-**	**Estimate**	**Judgment**
H1a: Past Experience→Risk Perception	+	0.430***	Yes
H1b: Past Experience→Behavioral Attitude	+	0.145**	Yes
H1c: Past Experience→Subjective Norms	+	0.181**	Yes
H1d: Past Experience→Willingness to Pay	+	0.140**	Yes
H2a: Risk Perception→Behavioral Attitude	+	0.143***	Yes
H2b: Risk Perception→Subjective Norms	+	0.013	No
H2c: Risk Perception→Perceived Behavioral Control	+	0.135**	Yes
H2d: Risk Perception→Willingness to Pay	+	0.055	No
H3a: Behavioral Attitude→Willingness to Pay	+	0.431***	Yes
H4a: Subjective Norms→Willingness to Pay	+	0.170***	Yes
H5a: Perceivedl Behavioral Control→Willingness to Pay	+	0.243***	Yes

*P < 0.05 denotes significant, **P < 0.01 denotes relatively significant, and ***P < 0.001 denotes very significant.

Risk perception was therefore shown to significantly affect behavioral attitudes (β = 0.143, *P* < 0.001) and perceived behavioral control (β = 0.135, *P* < 0.01). Therefore, hypothesis H2a and H2c are shown to be tenable, but they are not statistically significant for subjective norms (β = 0.013, *P* > 0.05) or willingness to pay (β = 0.055, *P* > 0.05), so H2b and H2d are not confirmed by the results. In addition, behavioral attitude (β = 0.431, *P* < 0.001), subjective norms (β = 0.170, *P* < 0.001) and perceived behavioral control (β = 0.243, *P* < 0.001) were shown to significantly affect WTP. Hypothesis H3a, H4a and H5a were therefore verified, which further verified the applicability of the theory of planned behavior.

When testing multiple mediating effects, Amos is only able to analyze the total indirect effects, but cannot analyze the indirect effects of specific variables of each mediating path. Therefore, the Bootstrap method (Preacher and Hayes, 2008) was used to test the mediating role of behavioral attitudes, perceived behavioral control, and subjective norms affecting both risk perception and willingness to pay, and the mediating roles of risk perception, behavioral attitude, and subjective norms between past experience and willingness to pay. These effect values were sorted by value, and 95% confidence intervals for mediating effects were estimated using the 2.5th and 97.5th percentiles. If the 95% confidence interval of the mediating effect did not include 0, this indicates that the mediating effect is significant. In this paper, we set a sample of 5,000 times and set a 95% confidence interval to get the specific indirect effect and confidence interval of each intermediary path. The results of this test are shown in Table 7.

**TABLE 7:** Bootstrap analysis results of the mediation effect test.

**Intermediary path**	**Indirect effect**	**BootSE**	**Bootstrapping 95% CI**	
			**LLCI**	**ULCI**
H2e: PE-RP-WTP	0.058	0.017	0.026	0.094
H3b: RP-BA-WTP	0.096	0.021	0.059	0.141
H3c: PE-BA-WTP	0.079	0.023	0.035	0.127
H4b: RP-SN-WTP	0.015	0.011	–0.006	0.037
H4c: PE-SN-WTP	0.038	0.015	0.011	0.068
H5b: RP-PBC-WTP	0.033	0.015	0.005	0.064

According to the criterion of whether the confidence interval contains 0 or not, we can see that risk perception as a mediating variable has a significant influence path on both willingness to pay and past experience [BC 95% CI = (0.026, 0.094)], assuming that H2e is true. Behavioral attitude is significant as a mediating variable in the path of risk perception [BC 95% CI = (0.059, 0.141)] and past experience [BC 95% CI = (0.035, 0.127)] on willingness to pay, assuming that H3b and H3c are true. The confidence interval of subjective norms as the mediating variable in the path of risk perception influencing willingness to pay [BC 95% CI = (−0.006, 0.037)] contains 0, and therefore the indirect effect of this variable is not significant, so H4b is not verified by this measure. However, subjective norms is significant as a mediating variable in the path of past experience influencing willingness to pay [BC 95% CI = (0.011, 0.068)], so H4c is shown to be verified. Finally, the confidence interval of perceived behavioral control as the mediating variable of risk perception’s influencing path on willingness to pay [BC 95% CI = (0.005, 0.064)] does not contain 0, and the mediating effect is tenable, and the hypothesis H5b is verified.

Given these results, we drew a table of the total effects, indirect effects, and direct effects of latent variables and willingness to pay (Table 8). According to Table 8, risk perception and past experience do not directly affect willingness to pay. Past experience affects willingness to pay through risk perception and subjective norms, and risk perception affects willingness to pay through subjective norms and perceived behavioral control.

**TABLE 8:** Direct effect, indirect effect, and total effect between latent variables and willingness to pay.

**Regression path**	**Direct effect**	**Indirect effect**	**Total effect**
Past Experience→Risk Perception	0.002	0.058	0.060
Past Experience→Behavioral Attitude	–	–	–
Past Experience→Subjective Norms	0.067	0.015	0.082
Past Experience→Willingness to Pay	–	–	–
Risk Perception→Behavioral Attitude	–	0.083	0.083
Risk Perception→Subjective Norms	0.085	0.015	0.100
Risk Perception→Perceived Behavioral Control	0.053	0.033	0.086
Risk Perception→Willingness to Pay	–	–	–
Behavioral Attitude→Willingness to Pay	0.087	–	0.087
Subjective Norms→Willingness to Pay	0.057	–	0.057
Perceived Behavioral Control→Willingness to Pay	0.058	–	0.058

*“–” means that the effect is not significant.*

**FIGURE 2:**
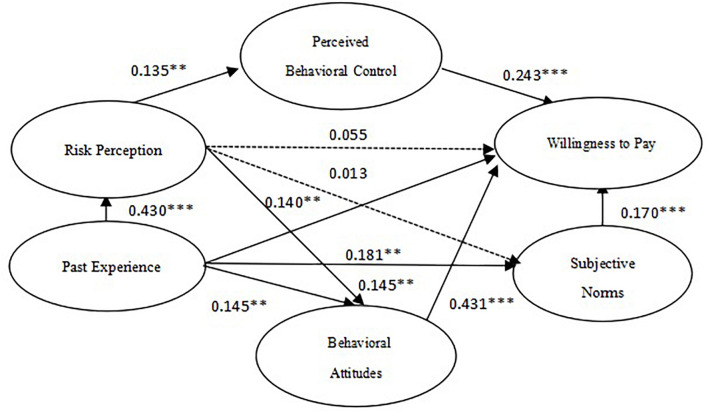
Results of structural model. *P < 0.1, **P < 0.05, and ***P < 0.01.

A summary overview of the impact model of urban residents’ willingness to pay for treatment of gutter oil is thereby shown in Figure 2.

### Difference Analysis Based on Socio-Economic Characteristics

We also included socio-economic characteristics in the influencing factors, and explored the impact of socio-economic characteristics on willingness to pay through one-way ANOVA and multiple groups of comparative analysis of structural equation models. The one-way ANOVA was produced using SPSS software to explore whether there are significant differences in variables across different groups. The multi group comparative analysis of the structural equation model can assume the differences in paths between different groups, and these differences can be measured by the AMOS software. Due to the limitation of the length of the paper, we only show the calculation results here. In the analysis of the difference of willingness to pay based on gender characteristics, the impact of risk perception on subjective norms is significant in female group, is not found to be significant in male groups, and the impact of behavioral attitude on willingness to pay is significant in the male group and is not significant in female group. In the difference analysis of willingness to pay based on age characteristics, middle-aged and younger people show a higher willingness to pay. The willingness to pay of respondents under the age of 50 was more than seven RMB, and the willingness to pay of respondents over the age of 50 was less than five RMB. In the difference analysis of willingness to pay based on income characteristics, high-income groups and low-income groups showed significant differences in behavioral attitude risk perception, subjective norms risk perception, and subjective norms on willingness to pay. The impact of behavioral attitude on willingness to pay was significant in the high-income group, and the impact of past experiences on subjective norms was significant in the low-income group; In the education-based differential analysis of willingness to pay, the equally significant paths of the low- and high-education groups include the impact of risk perception on behavioral attitude, subjective norms and perceived behavior control, subjective norms on willingness to pay, past experience on risk perception and subjective norms. The impact of behavioral attitude on willingness to pay was significant in the high-education group, and the influence of past experience on behavioral attitude was also significant in this group.

## Discussion and Implications

### Conclusion and Discussion

Most previous studies on this topic only calculated the willingness to pay, but did not explore the specific related factors and influence paths of the willingness to pay. This paper added two variables: past experience and risk perception. The theoretical framework of extended TPB was used to study the impact mechanism of the willingness of urban residents’ to pay for the treatment of gutter oil. The conclusions of this study can provide references for the making of relevant policy decisions.

Firstly, urban residents have a higher willingness to pay for treatment of gutter oil. Urban residents’ willingness to pay for treatment of gutter oil is 7.75 yuan, which is close to urban residents’ standard domestic garbage disposal fees (8 yuan per household per month). Nearly 80% of the respondents are willing to adhere to the principle of “the polluter pays” and to pay for a certain amount for the treatment of gutter oil. Consequently, they have a high willingness to pay. This means that urban residents have a high awareness of environmental protection, and also provides good opportunities for the expanded treatment of gutter oil in China.

Secondly, the hypotheses involved in the basic theoretical framework of the TPB show a good fit for the collected data. Behavioral attitudes, subjective norms and perceived behavioral control were found to significantly affect WTP, which is consistent with the TPB framework and the research conclusions of many scholars (Chen et al., 2016; Blaise et al., 2018; Kumar, 2019). This means that urban residents have a positive attitude toward the treatment of gutter oil, and show a certain degree of recognition and trust toward the process. Therefore, they are willing to pay a certain amount for the treatment of gutter oil. The variable of past experience added in the TPB framework achieves a good match for theoretical framework. The results show that residents’ past experience have a great impact on their risk perception (β = 0.430, *P* < 0.001), and also have a direct positive impact on behavioral attitude and subjective norms (Forward, 2009; Gardner, 2009). However, one unexpected finding was that residents’ risk perception showed no significant impact on their willingness to pay or subjective norms, which is inconsistent with the conclusions of some scholars (Covello et al., 2001; Simsekoglu, 2019). This paper believes that the reason for this result is that most humans cannot resist the temptation of food. For example, from past experience, people know that food with high fat will cause human obesity and cardiovascular disease (risk perception), but so many people still eat high fat food, especially hot pot. Although gutter oil is a waste and not a food, people eat gutter oil through food. Perhaps this is the reason why the risk perception of the gutter oil cannot have a significant impact on the willingness to pay and subjective norms.

Finally, results of the mediation effect test show that only one of the six paths of mediation effect in the structural equation model has not been verified. Residents’ risk perception and past experience indirectly affect their willingness to pay for the treatment of gutter oil through the intermediary variable of behavioral attitude, which means that public risk awareness can be improved by vigorously publicizing the harmful effects of poor or non-existent gutter oil treatment, so as to improve residents’ behavioral attitude toward the treatment of gutter oil. As a mediating variable, subjective norms have significant indirect effects on the influence path of past experience on willingness to pay, which reflects the important influence of subjective normative factors such as reference group and environment (Yadav and Pathak, 2017; Singh et al., 2018). Therefore, attention should be paid to the critical role of subjective norms. Meanwhile, risk perception was found to be a significant mediating variable in the influence path of past experience on willingness to pay, and perceived behavioral control was significant as a mediating variable in the influence path of risk control on willingness to pay. However, subjective norms were not a significant mediator in the impact of risk perception on willingness to pay.

### Implications

First, the government should establish special funding channels for gutter oil recovery. In accordance with the research conclusions, the residents’ willingness to pay for treatment of gutter oil averaged to 7.75 yuan, which is close to urban residents’ existing standard charge for garbage treatment. It can be seen that urban residents show a relatively high willingness to pay and sense of social responsibility. Given these conditions, the government can establish a reasonable payment mechanism for the service and set up special funds to support it. The main purpose of these special funds is to provide financial subsidies for gutter oil recycling and treatment enterprises. Gutter oil recycling and treatment enterprises can provide free recycling and treatment services for residents, which is important as these services actually involve significant labor costs, transportation costs, and operating costs. Therefore, special funds should be focused on well-regulated and authorized gutter oil recycling and treatment enterprises to support them to better provide services related to the recycling and treatment of gutter oil. The government can also collect appropriately rated fees from residents according to the “polluter pays” principle. The collected funds can then be used to install oil-water separators for household kitchens to better facilitate the recovery and treatment by associated enterprises. The government can also use these special funds to help enterprises to improve their scientific and technological innovation capabilities and enable them to develop more new technologies for treating gutter oil.

Second, investments should be made in storage equipment and the tracking and management of transport routes. It can be seen from the research conclusions that residents have a rather high willingness to pay for these services. Therefore, some cities can be selected for initial pilot projects to install oil-water separators in urban households and special storage equipment in residential areas, and to instruct residents to intentionally and autonomously put this filtered gutter oil into these special storage containers. As the residential gutter oil discharged locations are very widely distributed, the staff at these recycling enterprises should measure and collect the gutter oil at each point regularly every day, establish collection and transportation accounts for managing them, and form a rational and reasonable transportation route to collect from all containers regularly. The government regulatory department should install GPS units on the oil collection vehicles used for recycling, so as to effectively prevent recycling personnel from reselling the gutter oil illegally.

Third, a diverse array of publicity and educational activities should be carried out. As can be seen from the research conclusions, behavioral attitudes and subjective norms are the main direct factors affecting residents’ willingness to pay. Publicity and education are still the preferred means to influence individuals’ attitude toward environmental protection and their attitude toward the government, to adjust the public’s environmental behavior, and to increase their attention and sense of urgency toward the issue of gutter oil. Therefore, the government should use a variety of methods and channels to carry out a broad publicity and education campaign, vigorously popularize a common awareness of the environmental pollution caused by the unregulated disposal, collection, and reuse of gutter oil, promote basic knowledge of edible oil identification and relevant legal knowledge. The goal of this campaign is to guide residents to proactively protect the environment and maintain healthier consumption habits, as well as to improve public awareness of health, food safety, and environmental protection concerns. Different means of public communications can be adopted for residents of different ages. For younger residents, we should popularize knowledge about the treatment of gutter oil by means of internet media, micro-blogs, and public WeChat accounts. For elderly and other residents who do not use social media, we should adopt a more multimedia and community-based approach to educate them about the treatment of gutter oil.

Finally, media exposure should increase and enhance public awareness of associated risks. People’s risk perception is an indirect factor affecting their willingness to pay for these services, therefore law enforcement departments should regularly and publicly announce problems related to the disposal and recycling of “gutter oil” and kitchen waste, vigorous supporting the news media in their timely reporting of relevant topics and stories, measures taken, and their progress and results, and should expose any food service enterprises or illegal manufacturing or sales facilities that illegally use, produce, or sell gutter oil, and thereby improve public awareness of the risks of using untreated gutter oil.

## Data Availability Statement

The raw data supporting the conclusions of this article will be made available by the authors, without undue reservation.

## Author Contributions

PC and LX: conceptualization. LX: methodology. GS: software and data curation. QY: resources. PC: writing—original draft preparation. PC and GS: writing—review and editing. All authors have read and agreed to the published version of the manuscript.

## Conflict of Interest

The authors declare that the research was conducted in the absence of any commercial or financial relationships that could be construed as a potential conflict of interest.

## Publisher’s Note

All claims expressed in this article are solely those of the authors and do not necessarily represent those of their affiliated organizations, or those of the publisher, the editors and the reviewers. Any product that may be evaluated in this article, or claim that may be made by its manufacturer, is not guaranteed or endorsed by the publisher.
